# Landau-Kleffner Syndrome: A Diagnostic Challenge

**DOI:** 10.7759/cureus.7182

**Published:** 2020-03-05

**Authors:** Mushtaq Ahmed, Ayesha Saleem, Saad Nasir, Madiha Ariff, Pulwasha Iftikhar

**Affiliations:** 1 Pediatrics, Civil Hospital Karachi, Dow University of Health Sciences, Karachi, PAK; 2 Internal Medicine, United Medical and Dental College/Creek General Hospital, Karachi, PAK; 3 Internal Medicine, Civil Hospital Karachi, Dow University of Health Sciences, Karachi, PAK; 4 Obstetrics and Gynecology, St. John's University, New York, USA

**Keywords:** landau-kleffner syndrome, acquired epileptic aphasia, pediatric seizure

## Abstract

The Landau-Kleffner syndrome (LKS), formerly known as acquired epileptic aphasia, is a rare syndrome that typically presents in early childhood with language regression and seizures. We report a case of LKS in an 7-year-old boy who presented with aggressive behavior, difficulty in maintaining posture, and language regression. Systemic examination, including neurological evaluation, was normal. Cerebrospinal fluid (CSF) analysis and magnetic resonance imaging (MRI) were normal. Electroencephalogram (EEG) showed abnormal findings associated with generalized seizure discharge during sleep with more spikes being noted in bilateral frontal and temporal regions. LKS was diagnosed and was treated with anticonvulsants and steroids. On follow-up, the child showed improvement in maintaining posture, was able to walk independently and had improved linguistic functions. This case adds another variant of LKS to the existing literature.

## Introduction

In 1957, Landau and Kleffner described an unusual child disorder which they initially named syndrome of acquired aphasia with a convulsive disorder, later changing it to their name. In this syndrome, there is gradual language regression after a period of normal development along with seizures, but there is usually no causal relationship [1]. Most cases present between 3-7 years of age, with male to female ratio being 2:1 [2]. This condition initially presents with language problems presenting as word deafness or auditory verbal agnosia (AVA), in which the affected individual is unable to comprehend speech.

Seizures occur in 70%-85% Landau-Kleffner syndrome (LKS) patients. The onset of seizures is between age 4-10 years and typically stops after age 15. Abnormal electroencephalogram (EEG) pattern of spike and wave during sleep often precedes language improvement. Behavioral changes are believed to be due to language impairment. There is no standard treatment regimen for LKS, and various treatment modalities are used. The treatment options include anticonvulsant drugs, steroids, adrenocorticotropic hormone (ACTH), ketogenic diet, immunoglobulins, and surgery if required. Early use of steroids or ACTH can relieve symptoms and normalize EEG in LKS [3]. The EEG is a basic component in LKS diagnosis, which typically shows abnormal epileptic discharges in children, while clinical seizures are observed in about 70% of the cases. EEG shows spike and wave discharges that are widespread, multifocal or shifting, mostly in the temporal region, sometimes unilateral and are particularly exacerbated during sleep [4].

## Case presentation

A 7-year-old male was brought to the pediatric outpatient department by his mother. He presented with aggressive and hyperactive behavior associated with frequent falling from sitting and standing posture and he had a loss of speech from five months. These symptoms progressed rapidly during the first month but had been static for four months. Initially, he had a slurred speech which progressed to difficulty in articulation. He was unable to sit and stand without support and was not able to express a need for food. He also developed difficulty in understanding and obeying commands. He was an active child previously and he was able to perform routine activities and there was no family history of childhood seizure disorder.

On general physical examination, the child had an average height and built with no dysmorphic facial features. He was restless and hyperactive in the bed and was restraint by his mother with the forward falling of the trunk while sitting. The vital signs were all normal. Anthropometric measurements were taken, and were as follows: height 116 cm (10th-25th percentile), weight 20 kgs (25th percentile), and occipitofrontal circumference (OFC) 50 cm (25th percentile). He was partly responsive to the mother with purposeless movements and no verbal response to a command. The sensory and motor examination was normal. Cranial nerves were grossly intact. Cerebellar signs and nystagmus were absent. On gait examination, he was unable to walk without support. With support, no particular gait pattern was found; mixed movements associated with high stepping, jumping, and ataxic gait were observed. Systemic examination was unremarkable.

**Figure 1:**
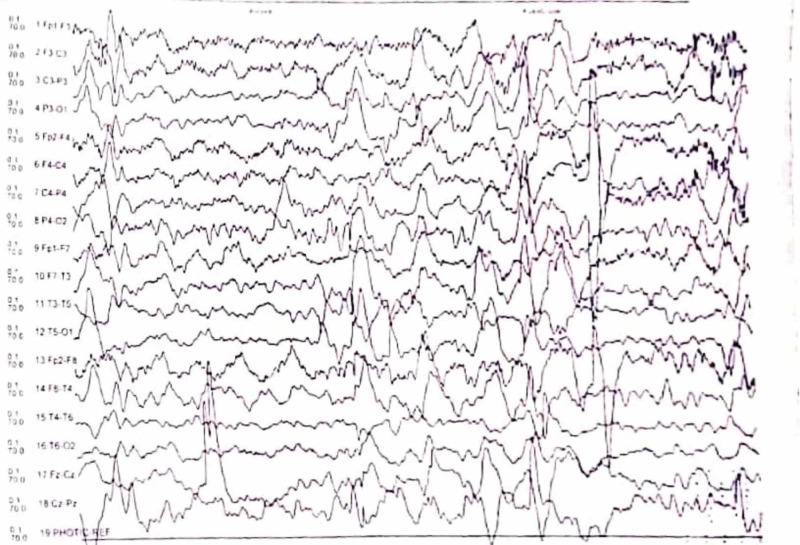
Electroencephalogram (EEG) during sleep showing spike and slow-wave discharge in a generalized manner

His laboratory investigations, including complete blood count, renal function tests, liver function tests were all normal. Cerebrospinal fluid (CSF) studies were normal. Magnetic resonance imaging (MRI) of the brain with contrast, appeared normal. EEG revealed abnormal findings of generalized seizure discharge with more spikes seen in bilateral frontal and temporal regions. During sleep, spike and slow-wave discharge were noted in both cerebral hemispheres in a generalized manner (Figure 1).

Based on clinical presentation and investigation, he was diagnosed as a case of LKS. After undergoing neurological and psychological consultations, treatment was started. The child was started on oral valproic acid and levetiracetam followed by clonazepam and haloperidol. The rapid clinical response associated with levetiracetam outweighed the possible risk of psychosis. There was no improvement in signs and symptoms, and he continued to have abnormal movements of limbs, frequent falling from sitting and standing posture. Oral prednisolone was started, and he showed significant clinical improvements in a month. His rehabilitation included speech therapy. There was an improvement in walking, and he was able to maintain his posture on his follow-up visit after three months of treatment. He was talking fluently, but his speech was still below average than his previously achieved level. He was able to play independently and was not aggressive but had some difficulty with other routine activities. He was continued on maintenance therapy with valproic acid, levetiracetam, and steroids.

## Discussion

Here, we discuss a case of LKS in a patient who presented to our outpatient department with complaints of aggressiveness and hyperactive behavior, and decline in speech along with an inability to maintain a sitting or standing posture. The differential diagnoses considered included subacute sclerosing panencephalitis, autism spectrum disorders (ASDs), myoclonic epilepsy and LKS as a diagnosis of exclusion. The LKS is often misdiagnosed as autism, pervasive developmental disorder, hearing impairment, dyslexia, auditory/verbal processing disorder, attention deficit disorder, intellectual disability, childhood schizophrenia, or emotional/behavioral problems in initial stages of presentation [5]. A case study by Kossoff et al. showed that the management of LKS patients who are symptomatic should include levetiracetam, as there is evidence suggesting that it halts the progression of the disease [6]. 

Motwani et al. reported an 11-year-old boy who presented with fever followed by convulsions [7]. The boy developed aphasia after his illness. His birth history was unremarkable, and he had normal growth and development of language, hearing, and vision. His neurological examination was normal. Investigations, including CSF study and MRI, were normal. However, EEG had an abnormal spike and wave exacerbation during sleep, and the boy was diagnosed as a case of LKS and treated with sodium valproate, levetiracetam and steroids [7]. In our case, the child had no history of fever preceding seizures or aphasia, and no clinically evident seizures, except for falling trunk movements and our child need more on the drugs to control the symptoms.

In another report by Raybarman, a 5-year-old boy developed aphasia, attention disorder, and hyperkinesia after a stressful event in his parents’ life [8]. EEG showed generalized epileptiform activity. He was diagnosed with LKS. Computed tomography (CT) scan and MRI of the brain were normal. He improved on antiepileptics and ACTH [8]. In our case, hyperkinetic and aggressive behavior was also noted, but the history of a traumatic or stressful event was absent.

Ghosh et al. also reported a case of a 9-year-old boy presented with loss of speech and seizure for two years [9]. The boy was developmentally normal before the onset of seizures. Examination of higher mental function revealed excessive hyperactivity, self-destructive behavior and decreased attention span. The audiological evaluation showed bilaterally normal hearing thresholds and he started communicating with signs. EEG showed repetitive spike and epileptiform activity from bilateral parieto-occipital regions. Clinical features and EEG led to a diagnosis of LKS [9]. This case is similar to ours, but the exceptional part of our case was the lack of clinical seizure activity.

There is scarce data available on LKS in the literature, and its a challenge to diagnose LKS, which leads to unnecessary treatment, financial burden, and undue stress to the patient.

## Conclusions

This case report highlight the fact that LKS can present with language regression along with aggressive behavior and difficulty in maintaining posture with an absence of seizure activity, making it hard to fit in the spectrum of LKS. A thorough evaluation must be done to rule out other causes, and EEG with sleep potentiation must be done in all cases. This case adds another variant of LKS to the existing literature.
